# Supplementary figures and images for: Windows of opportunity for daily physical activity
Source: PLoS One. 2020 Sep 23;15(9):e0238713. doi: 10.1371/journal.pone.0238713 (PMC7510972; doi:10.1371/journal.pone.0238713)

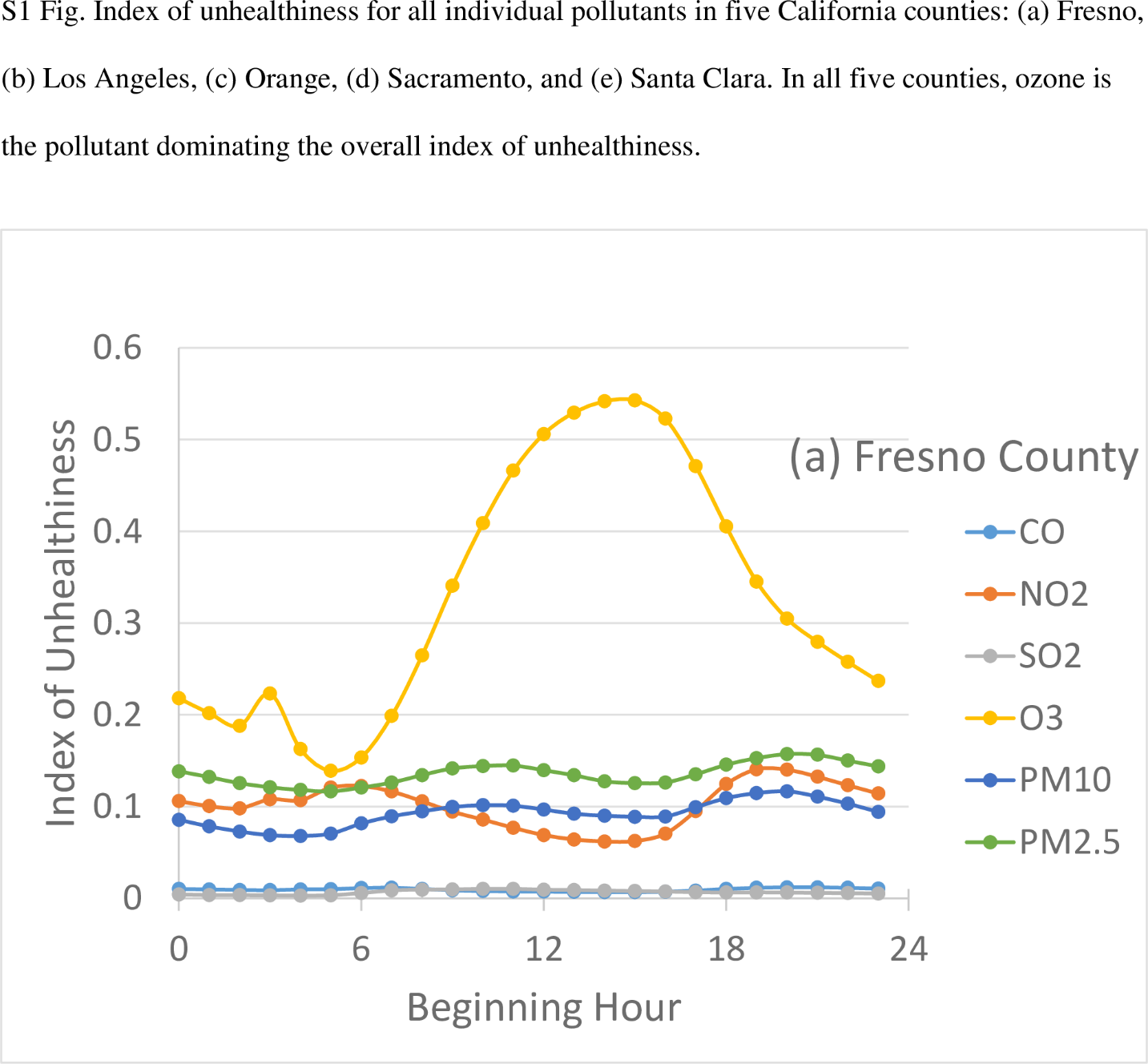

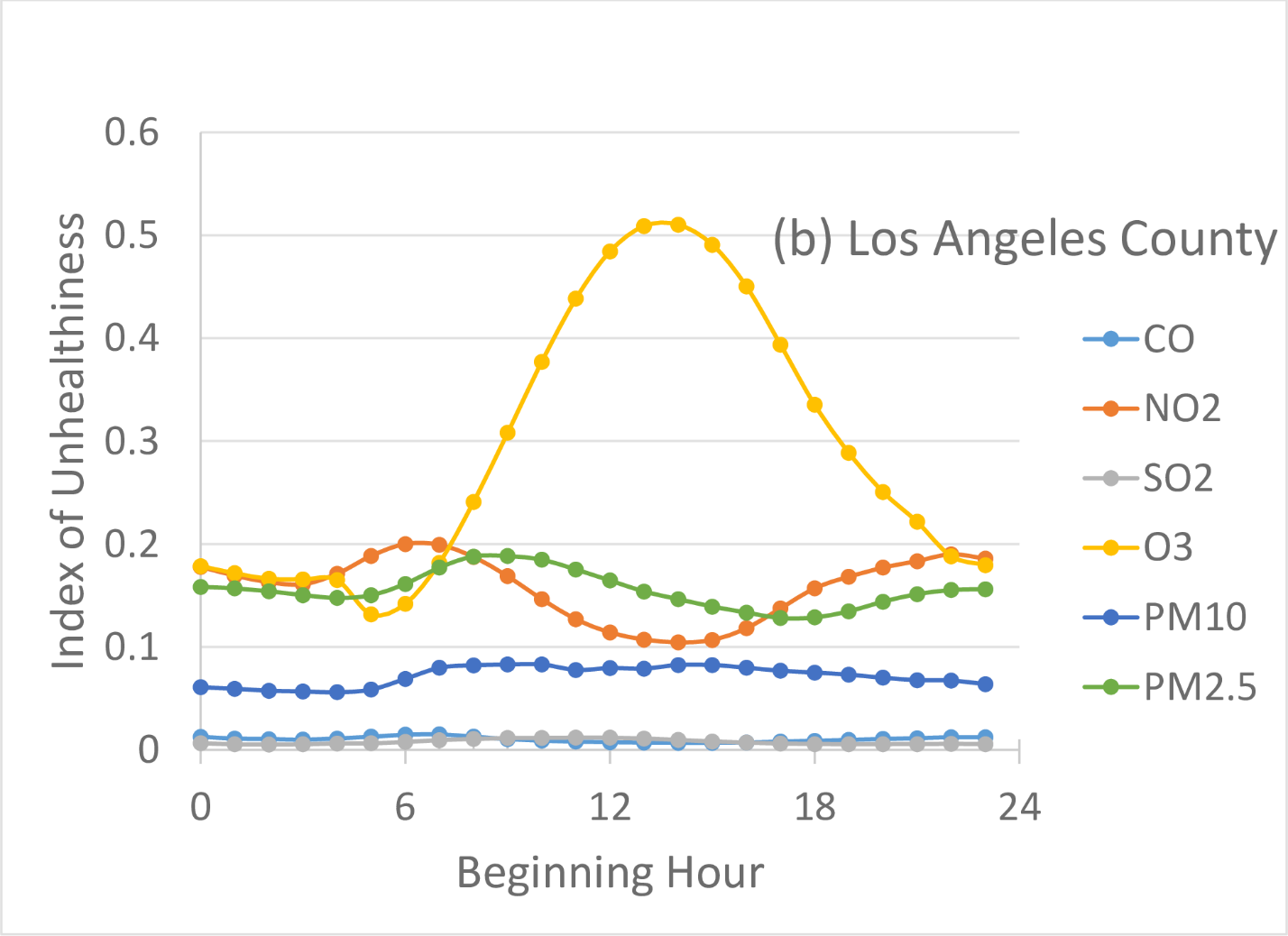

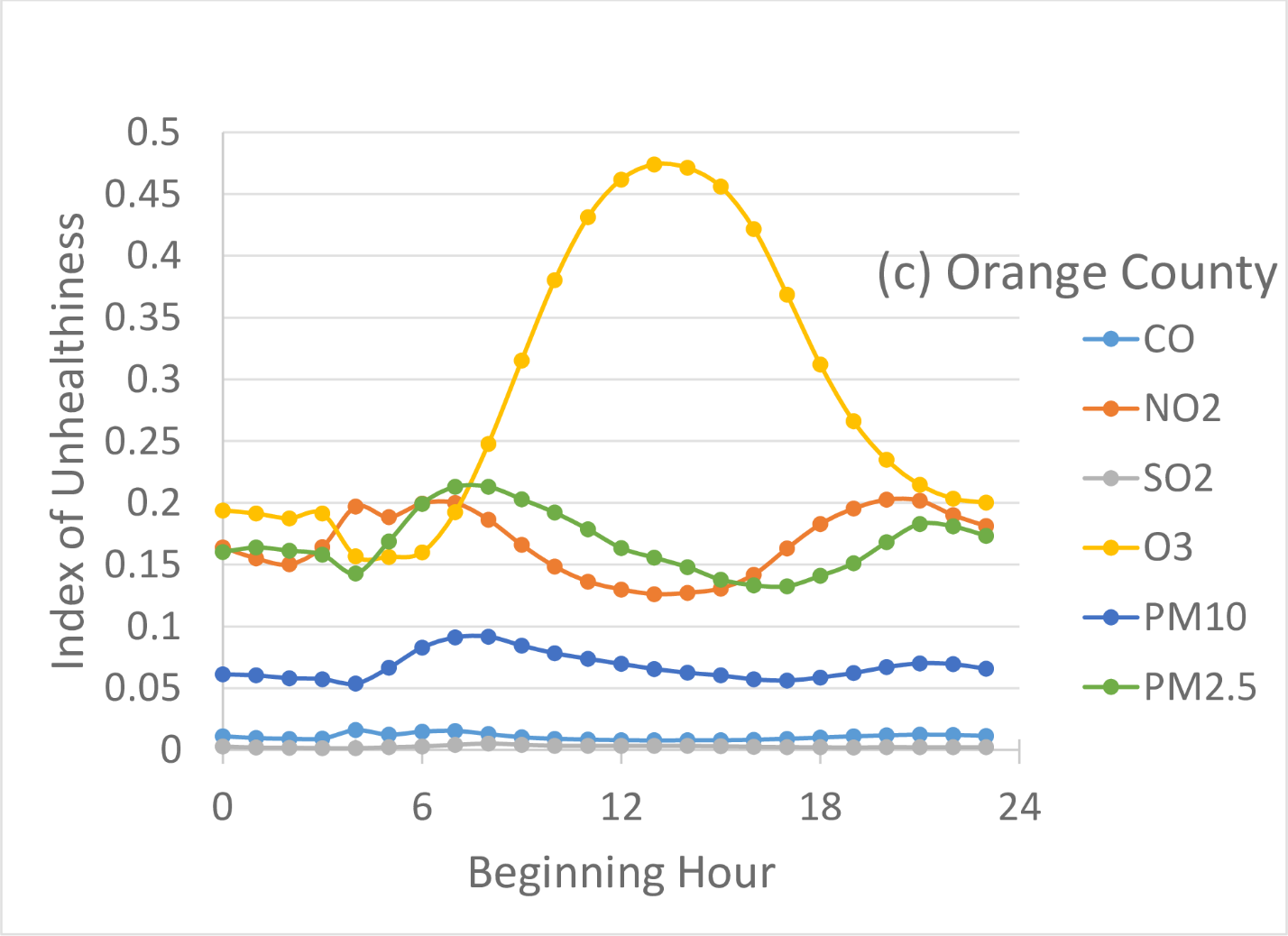


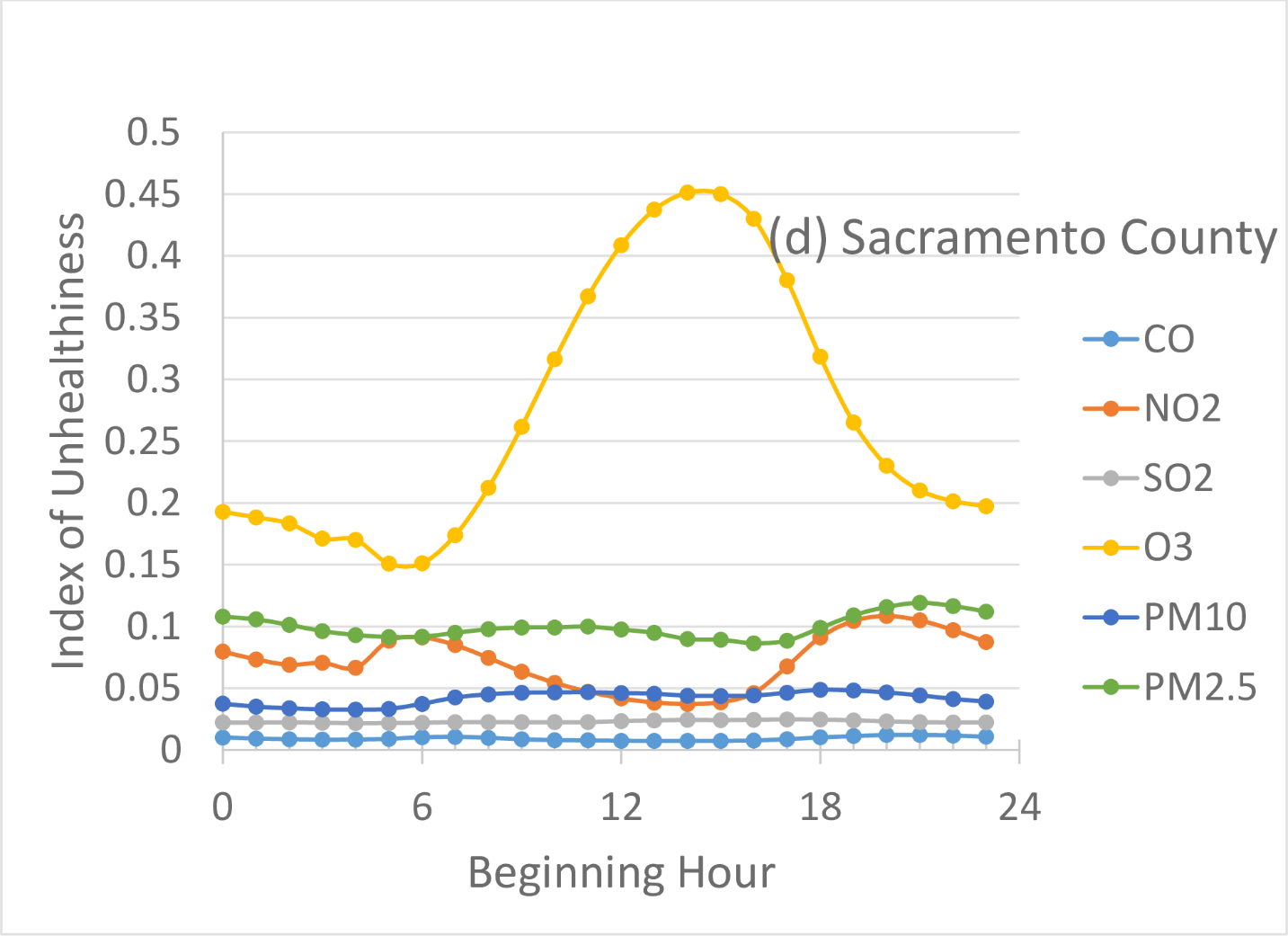

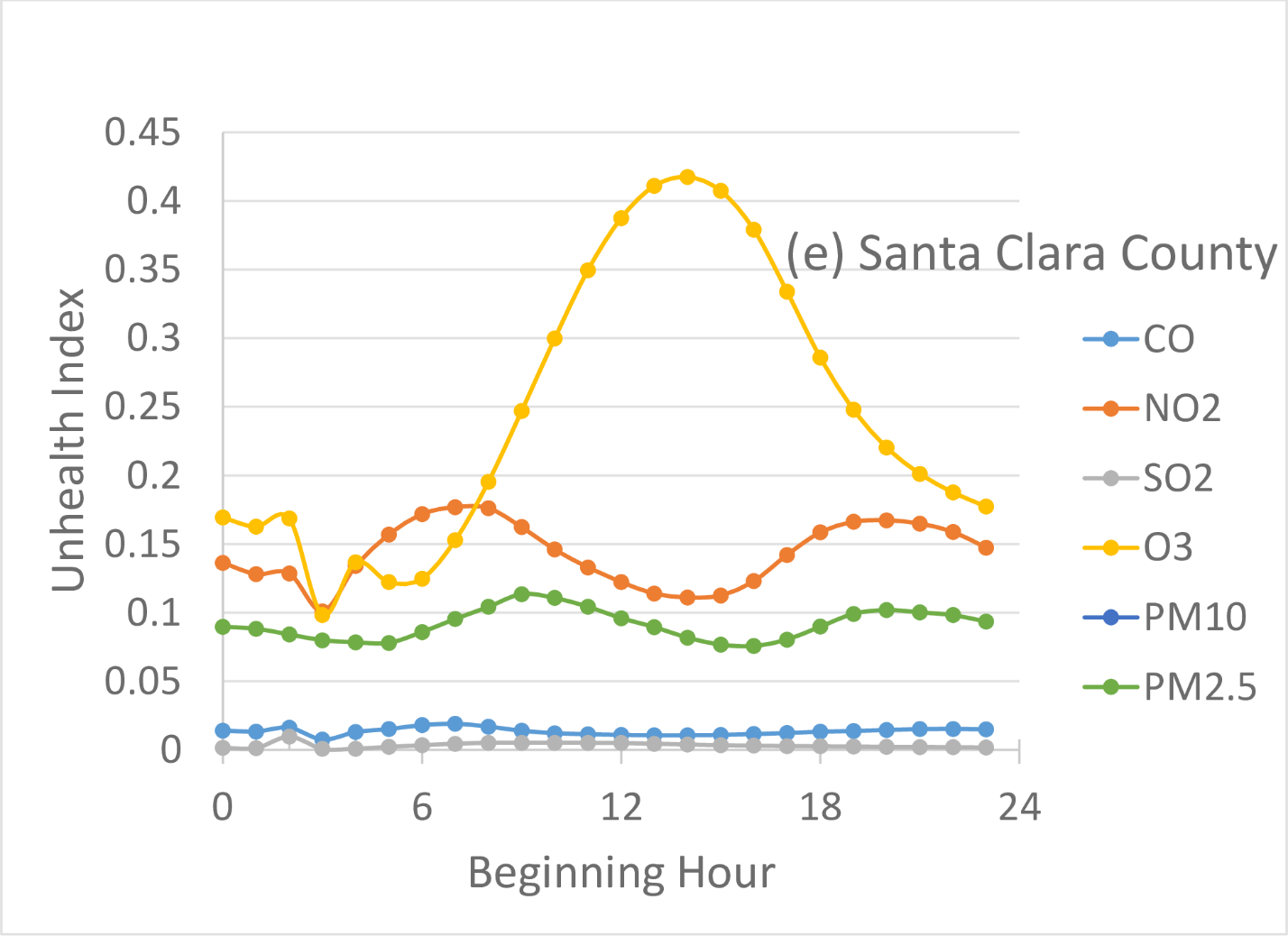

Supplement: S1 Fig — (a) Fresno, (b) Los Angeles, (c) Orange, (d) Sacramento, and (e) Santa Clara. In all five counties, ozone is the pollutant dominating the overall index of unhealthiness. (DOCX) [file pone.0238713.s001.docx]

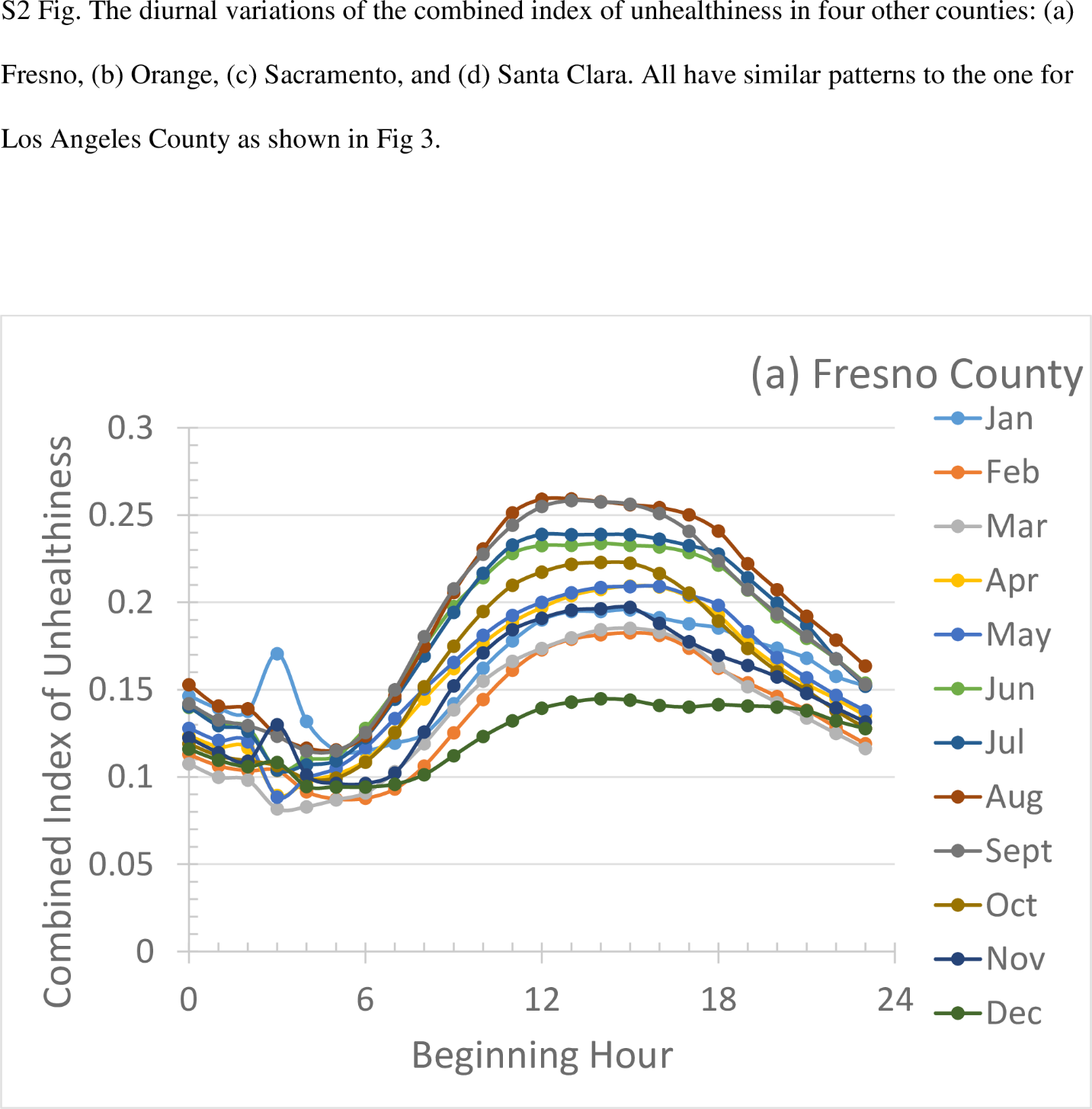

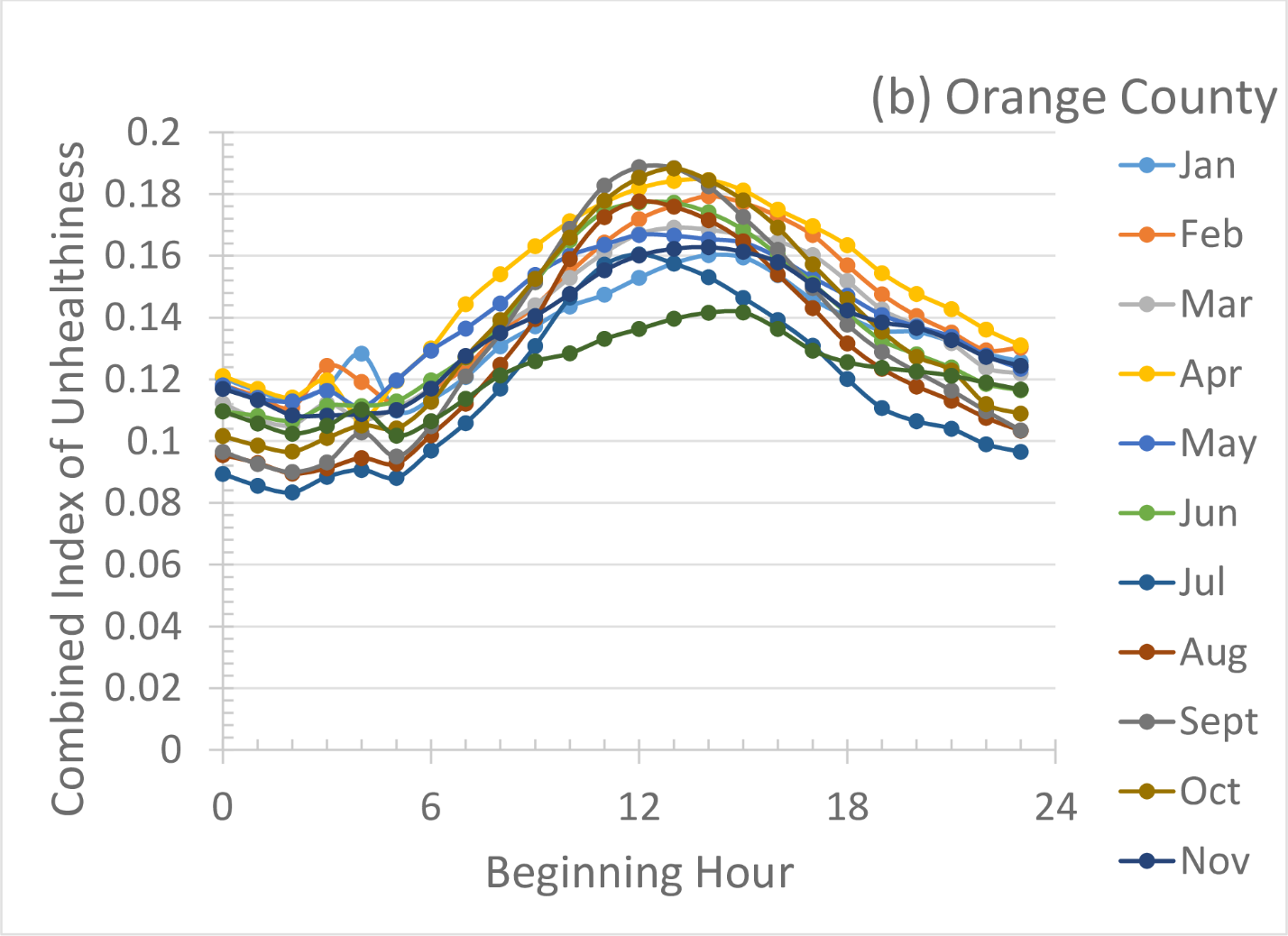

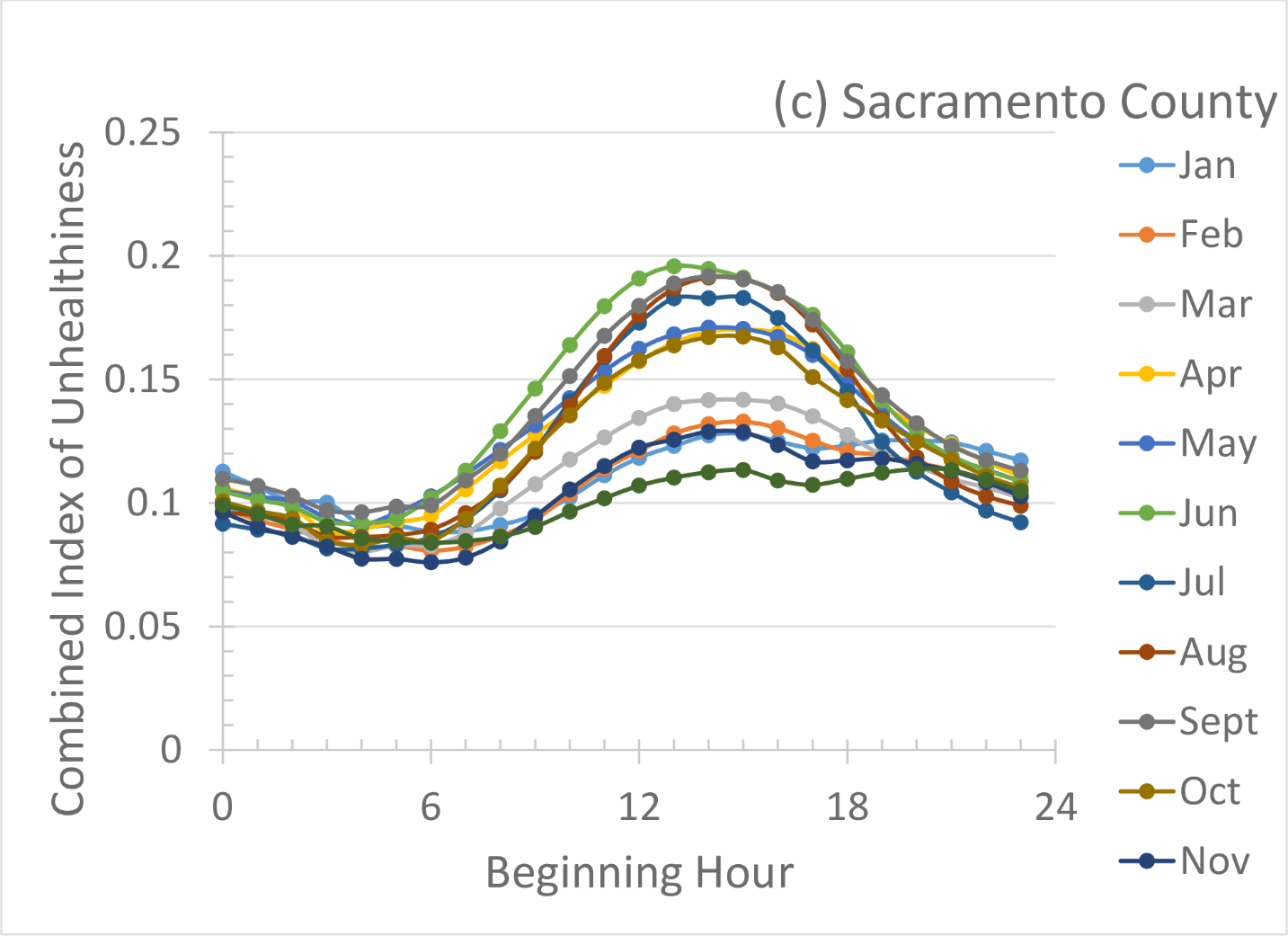

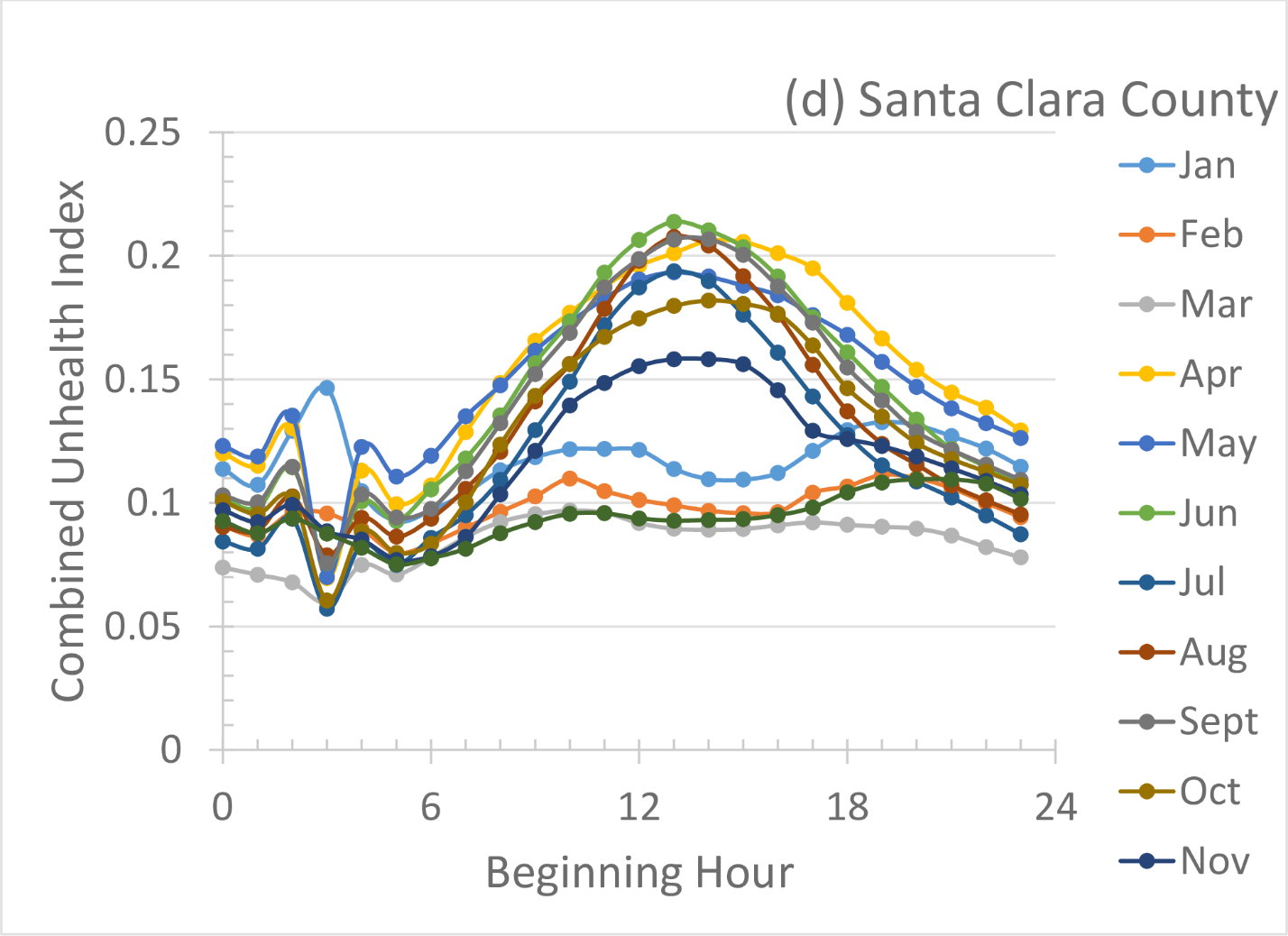

Supplement: S2 Fig — (a) Fresno, (b) Orange, (c) Sacramento, and (d) Santa Clara. All have similar patterns to the one for Los Angeles County as shown in Fig 3. (DOCX) [file pone.0238713.s002.docx]
