# Supplementary material for: Windows of opportunity for daily physical activity
Source: PLoS One. 2020 Sep 23;15(9):e0238713. doi: 10.1371/journal.pone.0238713 (PMC7510972; doi:10.1371/journal.pone.0238713)
Supplement: Attachment — Submitted filename: Report PLOS ONE.pdf [file pone.0238713.s003.pdf]

Referee's report of “Windows of Opportunity for Daily Physical Activity”

In the note by Marvin Du, entitled: ”Windows of Opportunity for Daily Physical Activity”, the author studies a method that can use air quality data in a large area to derive a diurnal profile of air quality variation for that area and to identify the time window that the air quality is typically the best throughout the day.

The paper is interesting, interesting technical work and innovative subject, beneficial and significant to study the complex problem to improve the air quality, since it possesses sociological and biological implications and extends the former researches. The questions posed are identifiable and understood, the conditions are well controlled, the interpretation discuss the relevance of all the results in a clear manner. The reference list follow the format for the journal, but could be boosted. The paper is a significant piece of mathematics, of interest to a broad section of the differential equations community, and it is a true advance.

According to all the motivations below, it is worthy of publication in *PLOS ONE*, but before it I suggest some minor essential modifications:

1) pg.1 line 10, for a better diffusion of the paper I suggest to add ”PM10” and ”PM25”;

2) pg.5 line 103, I kindly request to explain why in (1) the one-hour national air quality standard is surely different from zero;

3) pg.5 line 106, I suggest to write ”in a month taking into account that N_d varies

between 28 and 31";

4) pg.6 line 127, is the pollutant-specific scaling factor different from zero, why ? Please, explain to the readers it;

5) pg.8 line 164, please, spend some words to explain, in your opinion, why the other pollutants stay relatively flat;

6) pg.9 line 183, I suggest write shortly Discussion and Conclusion, because could be useful for the reader, briefly understand, especially, what the present analysis can gives as conclusion. In this sense, I suggest to delete the initial part and do not repeat more than one time the same conclusion, for example, that the study is valid just in California.
