# Supplementary material for: Windows of opportunity for daily physical activity
Source: PLoS One. 2020 Sep 23;15(9):e0238713. doi: 10.1371/journal.pone.0238713 (PMC7510972; doi:10.1371/journal.pone.0238713)
Supplement: Attachment — Submitted filename: REPORT_n_2_in_the_right_LINE.pdf [file pone.0238713.s004.pdf]

**Reviewer Recommendation Term:**	Minor Revision
**Rate Review:**	0
**Custom Review Question(s):**	**Response**
**Comments to the Author**<br><br>1. Is the manuscript technically sound, and do the data support the conclusions?<br><br>The manuscript must describe a technically sound piece of scientific research with data that supports the conclusions. Experiments must have been conducted rigorously, with appropriate controls, replication, and sample sizes. The conclusions must be drawn appropriately based on the data presented.	
2. Has the statistical analysis been performed appropriately and rigorously?	
3. Have the authors made all data underlying the findings in their manuscript fully available?<br><br>The PLOS Data policy requires authors to make all data underlying the findings described in their manuscript fully available without restriction, with rare exception (please refer to the Data Availability Statement in the manuscript PDF file). The data should be provided as part of the manuscript or its supporting information, or deposited to a public repository. For example, in addition to summary statistics, the data points behind means, medians and variance measures should be available. If there are restrictions on publicly sharing data—e.g. participant privacy or use of data from a third party—those must be specified.	
4. Is the manuscript presented in an intelligible fashion and written in standard English?<br><br>PLOS ONE does not copyedit accepted manuscripts, so the language in submitted articles must be clear, correct, and unambiguous. Any typographical or grammatical errors should be corrected at revision, so please note any specific errors here.	
5. Review Comments to the Author<br><br>Please use the space provided to explain your answers to the questions above. You may also include additional comments for the author, including concerns about dual publication, research ethics, or publication ethics. (Please upload your review as an attachment if it exceeds 20,000 characters)	The paper, focused on the correlation between air pollution and health benefit is quite specific and in the meantime not useful for a lots of readers of the journal, the argument does involves a very big area and, probably, the journal PLOS ONE is the best choice for it.<br>I suggest, before publication, to:<br>1. read again the paper and remove some english misprints,<br>2. put the references of the bibliography the same characters,<br>3. pg.2 line 32, write ��A comprehensive study [3] (see also e.g. [X] and reference therein)��, where :<br>[X] Rapisarda V, Loreto C, Vitale E, Matera S, Ragusa R, Coco G, Rapisarda L, Ledda C, 2019, ��Incidence of sharp and needle-stick injuries and mucocutaneous blood exposure among healthcare workers��, Future Microbiology 14 (9), 27-31, Supplement: S, doi: 10.2217/fmb-2018-0239,<br>4. pg.38 line 2 delete ��show��.<br>With that, I recommend the paper to be accepted after minor revision.
6. PLOS authors have the option to publish the peer review history of their article (what does this mean?). If published, this will include your full peer review and any attached files.<br><br>If you choose "no", your identity will remain anonymous but your review may still be made public.<br><br>**Do you want your identity to be public for this peer review?** For information about this choice, including consent withdrawal, please see our Privacy Policy.	None
**Confidential to Editor**<br><br>1. Do you have any potential or perceived competing interests that may influence your review? Please review our Competing Interests policy and declare any potential interests that you feel the Editor should be aware of when considering your review. If you have no competing interests, please write "I have no competing interests."	
2. Did you receive any assistance in preparing this review (e.g. from a post-doc or graduate student)? If yes, please include their name below.	
3. If accepted, do you think this submission should be highlighted on the PLOS ONE website? PLOS ONE does not evaluate manuscripts based on perceived significance or readership. We aim to provide tools for readers to filter and evaluate our publications. (optional)	

More Reviewer Details

Cancel Save and Close
