# Supplementary material for: Windows of opportunity for daily physical activity
Source: PLoS One. 2020 Sep 23;15(9):e0238713. doi: 10.1371/journal.pone.0238713 (PMC7510972; doi:10.1371/journal.pone.0238713)
Supplement: Attachment — Submitted filename: Responses to reviewers.docx [file pone.0238713.s005.docx]

All the responses are in boldface.

Response to the Academic Editor:

1. Please ensure that your manuscript meets PLOS ONE's style requirements, including those for file naming. The PLOS ONE style templates can be found at

https://journals.plos.org/plosone/s/file?id=wjVg/PLOSOne_formatting_sample_main_body.pdf and

<https://journals.plos.org/plosone/s/file?id=ba62/PLOSOne_formatting_sample_title_authors_affiliations.pdf>

**Response: The revised manuscript has followed the direction.**

 2. Please ensure that you include a title page within your main document. We do appreciate that you have a title page document uploaded as a separate file, however, as per our author guidelines (http://journals.plos.org/plosone/s/submission-guidelines#loc-title-page) we do require this to be part of the manuscript file itself and not uploaded separately.
Could you therefore please include the title page into the beginning of your manuscript file itself, listing all authors and affiliations?

**Response: The revised manuscript has followed the direction.**

3. In your Data Availability statement, you have not specified where the minimal data set underlying the results described in your manuscript can be found. PLOS defines a study's minimal data set as the underlying data used to reach the conclusions drawn in the manuscript and any additional data required to replicate the reported study findings in their entirety. All PLOS journals require that the minimal data set be made fully available. For more information about our data policy, please see http://journals.plos.org/plosone/s/data-availability.

Upon re-submitting your revised manuscript, please upload your study’s minimal underlying data set as either Supporting Information files or to a stable, public repository and include the relevant URLs, DOIs, or accession numbers within your revised cover letter. For a list of acceptable repositories, please see http://journals.plos.org/plosone/s/data-availability#loc-recommended-repositories. Any potentially identifying patient information must be fully anonymized.

Important: If there are ethical or legal restrictions to sharing your data publicly, please explain these restrictions in detail. Please see our guidelines for more information on what we consider unacceptable restrictions to publicly sharing data: http://journals.plos.org/plosone/s/data-availability#loc-unacceptable-data-access-restrictions. Note that it is not acceptable for the authors to be the sole named individuals responsible for ensuring data access.

We will update your Data Availability statement to reflect the information you provide in your cover letter.

**Response: The cover letter has information for the availability of the datasets used in the paper.**

4. Please include copies of Tables 1 and 2 which you refer to in your text on pages 5 and 6.

**Response: The two tables have been added to the manuscript.**

5. Please include captions for your Supporting Information files at the end of your manuscript, and update any in-text citations to match accordingly. Please see our Supporting Information guidelines for more information: [http://journals.plos.org/plosone/s/supporting-information](about:blank).

**Response: The revision has followed the direction.**

Response to reviewer #1:

1) pg.1 line 10, for a better diffusion of the paper I suggest to add "PM10" and

"PM25";

**Response: The change has been made.**

2) pg.5 line 103, I kindly request to explain why in (1) the one-hour national air

quality standard is surely different from zero;

**Response: A clarification has been added.**

3) pg.5 line 106, I suggest to write "in a month taking into account that Nd varies

1

between 28 and 31";

**Response: The change has been made.**

4) pg.6 line 127, is the pollutant-specific scaling factor different from zero, why ?

Please, explain to the readers it;

**Response: A clarification has been added.**

5) pg.8 line 164, please, spend some words to explain, in your opinion, why the other

pollutants stay relatively at;

**Response: The change has been made.**

6) pg.9 line 183, I suggest write shortly Discussion and Conclusion, because could be

useful for the reader, briey understand, especially, what the present analysis can gives

as conclusion. In this sense, I suggest to delete the initial part and do not repeat more

than one time the same conclusion, for example, that the study is valid just in California.

**Response: Some of the redundant wording has been deleted.**

Response to Reviewer #2:

1) Read again the paper and remove some English misprints.

**Response: Some typos have been removed.**

2) Put the reference of the bibliography the same characters.

**Response: The change has been made.**

3) Pg. 2 line 32, write “A comprehensive study”

**Response: The change has been made.**

4) Pg. 2, line 38. Delete “show”

**Response: The word has been deleted.**
