# Supplementary material for: Windows of opportunity for daily physical activity
Source: PLoS One. 2020 Sep 23;15(9):e0238713. doi: 10.1371/journal.pone.0238713 (PMC7510972; doi:10.1371/journal.pone.0238713)
Supplement: Attachment — Submitted filename: Responses to reviewers_email.docx [file pone.0238713.s006.docx]

Response to reviewer #1:

1) pg.1 line 10, for a better diffusion of the paper I suggest to add "PM10" and

"PM25";

**Response: The change has been made in ‘Revised manuscript with track changes’ page 3 lines 44-45.**

2) pg.5 line 103, I kindly request to explain why in (1) the one-hour national air

quality standard is surely different from zero;

**Response: A clarification has been added in ‘Revised manuscript with track changes’ page 7 lines 129-130.**

3) pg.5 line 106, I suggest to write "in a month taking into account that Nd varies

1

between 28 and 31";

**Response: The change has been made in ‘Revised manuscript with track changes’ page 7 lines 126-127.**

**.**

4) pg.6 line 127, is the pollutant-specific scaling factor different from zero, why ?

Please, explain to the readers it;

**Response: A clarification has been added in ‘Revised manuscript with track changes’ page 8 lines 149-150.**

**.**

5) pg.8 line 164, please, spend some words to explain, in your opinion, why the other

pollutants stay relatively at;

**Response: The change has been made in ‘Revised manuscript with track changes’ pages 9-10 lines 185-186. It is hard to give a more specific and clearer explanation because most of the time concentrations of pollutants change with wind speed and mixing height. Because wind speed and mixing height have strong variations with hour of day, so concentrations of pollutants can certainly vary with hour of day. The reason I make a relatively lengthy explanation here rather than in the manuscript is that the explanation is somewhat cumbersome.**

6) pg.9 line 183, I suggest write shortly Discussion and Conclusion, because could be

useful for the reader, briefly understand, especially, what the present analysis can gives

as conclusion. In this sense, I suggest to delete the initial part and do not repeat more

than one time the same conclusion, for example, that the study is valid just in California.

**Response: Some of the redundant wording has been deleted in ‘Revised manuscript with track changes’ page 11 lines 211-213. Line 226-228 were also deleted.**

Response to Reviewer #2:

1) Read again the paper and remove some English misprints.

**Response: Some typos have been removed. This is shown in numerous pages at multiple places.**

2) Put the reference of the bibliography the same characters.

**Response: The change has been made in ‘Revised manuscript with track changes’ pages 14-15 lines 269-310.**

3) Pg. 2 line 32, write “A comprehensive study [3] (see also e.g. Rapisarda V. et al. …

**Response: This is the only comment that I was not able to fully address. This reason is as follows. It is not clear to me if the reviewer suggested to add just the words ‘and reference therein’ or actually add the reference by Rapisarda et al. If it is the former, I did add the words in ‘Revised manuscript with track changes’ page 3 line 51; if it is the latter, I am afraid I cannot add the paper in the reference list because I read the paper and found it not related to my work. I wonder if the reviewer meant a different paper.**

4) Pg. 2, line 38. Delete “show”

**Response: The word has been deleted in ‘Revised manuscript with track changes’ page 3 line 57.**
